# Compact and Wide-Stopband Bandpass Filter Using Hybrid Shielded EMCSIW and CSRR Resonators with a Mixed Electromagnetic Coupling Scheme

**DOI:** 10.3390/mi15121426

**Published:** 2024-11-27

**Authors:** Zhuo-Wei Miao

**Affiliations:** State Key Laboratory of Millimeter-Waves, School of Information Science and Engineering, Southeast University, Nanjing 210096, China; zwmiao@seu.edu.cn

**Keywords:** bandpass filter (BPF), complementary split ring resonator (CSRR), shielded eighth-mode circular substrate-integrated waveguide (SD-EMCSIW), mixed coupling

## Abstract

This paper presents a bandpass filter (BPF) exploiting hybrid shielded eighth-mode circular substrate-integrated waveguide (SD-EMCSIW) and complementary split ring resonator (CSRR) resonators. The proposed BPF leverages the SD-EMCSIW resonator with a 45-degree angle to create a second-order BPF with a mixed electromagnetic coupling scheme. Detailed analyses of the related electromagnetic characteristics and operating mechanisms have been performed. In order to further reduce the occupied area, the CSRR structures are embedded into the SD-EMCSIW resonators. Meanwhile, extra metallic via-holes are implemented to enhance the upper-stopband performance. A transmission zero (TZ) of the second-order BPF can be placed on either the left or right side of the passband and can be flexibly adjusted. To validate the design concept, a second-order hybrid SD-EMCSIW and CSRR BPF was designed, simulated, fabricated, and measured as a specific example. The prototype operates at a center frequency *f*_0_ of 8.3 GHz with a 3 dB fractional bandwidth of 8.1%. Two transmission zeros are located near the right passband. The upper-stopband rejection reaches up to 15 dB at 2.85 times the center frequency *f*_0_. Both the simulated and measured results show satisfactory agreement. Meanwhile, the overall size of the proposed hybrid SD-EMCSIW and CSRR BPF is 13.5 mm × 13.0 mm (0.37*λ*_0_ × 0.36*λ*_0_), featuring a compact physical dimension in the filter design.

## 1. Introduction

Recently, the bandpass filter (BPF) has emerged as one of the most critical components in contemporary radar and wireless communication systems [1,2]. These filters demand stringent criteria for low cost, small size, and excellent performance. Different types of resonators are used in designing these filters, including metallic waveguide [3], dielectric resonator (DR) [4], microstrip line (MSL) [5], slot-line [6], and substrate-integrated waveguides (SIWs) [7,8,9,10,11]. Among these, SIW-based BPFs are favored for their appealing attributes, such as low loss, low cost, planar construction, and ease of fabrication. To reduce the dimensions of SIW BPFs, sub-mode approaches have been implemented. Examples include half-mode SIW (HMSIW) BPFs [12,13], quarter-mode SIW (QMSIW) BPFs [14,15], eighth-mode SIW (EMSIW) BPFs [16,17], and sixteenth-mode SIW (SMSIW) BPFs [18,19]. Most miniaturized SIW resonators currently have a rectangular design, whereas circular structures are less prevalent. However, the design flexibility and freedom of rectangular SIW (RSIW) cavities fall short compared to circular SIW (CSIW) cavities [20,21,22,23,24]. Additionally, the shielded SIW (SD-SIW) resonators have been investigated in several bandpass filter designs, accounting for their excellent quality-factor and shield properties [25,26,27]. Furthermore, there has not yet been a comprehensive study on shielded circular substrate-integrated waveguide (SD-CSIW) resonators and their application in bandpass filters with mixed coupling. In addition to frequency selectivity and design complexity, physical footprints and stopband rejection are also vital metrics for evaluating BPF performance. Thus, it is imperative to develop a high-performance BPF that integrates compact size, high selectivity, extensive stopband suppression, and simplicity, ensuring practical utility.

In this paper, a compact-sized and wide-stopband SD-EMCSIW and CSRR BPF has been introduced. The SD-EMCSIW resonator has been studied systematically and in detail. Additionally, a second-order SD-EMCSIW BPF featuring mixed electromagnetic coupling has been proposed. A transmission zero can be generated on the right side of the passband, and this transmission zero can be adjusted flexibly. Moreover, the proposed second-order BPF displays wide-stopband performance. Furthermore, a second-order hybrid EM CSIW and CSRR BPF with a mixed electromagnetic coupling topology has been integrated into a single-layer circuit board, showcasing a simple structure, high selectivity, compact design, and wide-stopband performance.

## 2. Analysis of the Shielded CSIW and CSRR Resonator

### 2.1. Analysis of Full-Mode and Sub-Mode CSIW Resonator

**Figure 1:**
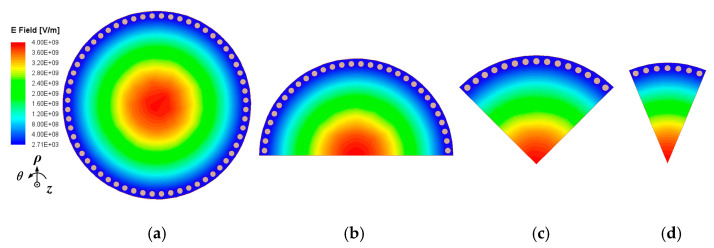
Configuration and electric-field distributions of full-mode and sub-mode CSIW resonators. (**a**) FMCSIW (**b**) HMCSIW (**c**) QMCSIW (**d**) EMCSIW.

The configurations of full-mode and sub-mode circular substrate-integrated waveguide (SMCSIW) resonators are depicted in Figure 1. The SMCSIW resonators display the layout of half-mode CSIW (HMCSIW), quarter-mode CSIW (QMCSIW), and eighth-mode CSIW (EMCSIW). In addition, the electric-field distributions of the resonators are illustrated. It can be observed that the fundamental mode of the first mode is TM_010_. The dimensional radius of the circular cavity is about 7.9 mm. As for the eigenmode simulation, the boundary conditions are perfect H and the frequency of the eigenmode is approximately 10 GHz.

The resonance frequencies of the TM modes in the full-mode CSIW can be derived through the following analytical method [28,29]:

(1) $$f_{TM_{nml}}=\frac{c}{2\pi \sqrt{\mu_{r}\varepsilon_{r}}}\sqrt{{\left(\frac{p_{nm}}{r}\right)}^{2}+{\left(\frac{\pi l}{h}\right)}^{2}}$$

where *f*_TM*nml*_ denotes the resonance frequency; *c* represents the speed of light in free space; and *n*, *m*, and *l* are the indices of the TM*_nml_* modes. Additionally, *r* and *h* represent the dimensional equivalent radius of the circular cavity and the thickness of the substrate, respectively. The variable *p_nm_* signifies the *m*-th root of the Bessel function of order *n*, and *r* indicates the effective radius of the CSIW resonator. Given that the substrate thickness is less than half the wavelength, the CSIW resonator supports only the resonance of TM_*nm*0_ modes. Consequently, the resonant frequency of full-mode CSIW resonator can be derived as:

(2) $$f_{TM_{nm0}}=\frac{c}{\sqrt{\varepsilon_{r}}}\frac{p_{nm}}{2\pi r}$$

Furthermore, the resonant frequency of the sub-mode CSIW resonator can be expressed by:

(3) $$f_{TM_{nm0}}=\frac{c}{\sqrt{\varepsilon_{r}}}\frac{\eta (n\pi /\theta ,m)}{2\pi r}$$

where *η*(*nπ*/*θ*, *m*) signifies the *m*-th root of the Bessel function of the first kind and fractional order of *nπ*/*θ*.

**Figure 2:**
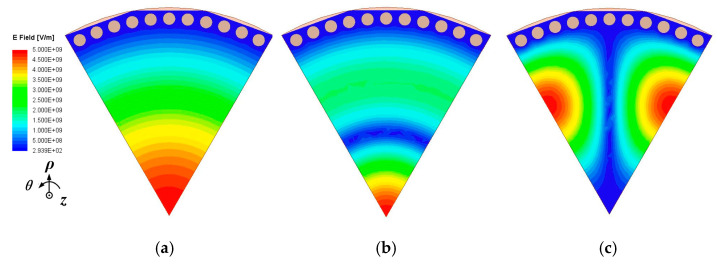
Electric-field distributions of the first three sub-mode CSIW modes: (**a**) TM_010_, (**b**) TM_020_, (**c**) TM_110_.

The relevant electric-field distributions of the first three modes in the SMCSIW resonator can be found in Figure 2. As can be observed, the first three modes are TM_010_, TM_020_ and TM_110_. It should be mentioned that the second and third modes of the SMCSIW resonant cavity could be TM_020_ or TM_110_. This depends on the angular range of the sub-mode SIW resonator. When the angle exceeds 77 degrees, the second high-order mode is TM_110_, and the third high-order mode is TM_020_. When the angle is less than 77 degrees, the sequence order of these two modes is reversed.

### 2.2. Analysis of Shielded Sub-Mode CSIW Resonator

**Figure 3:**
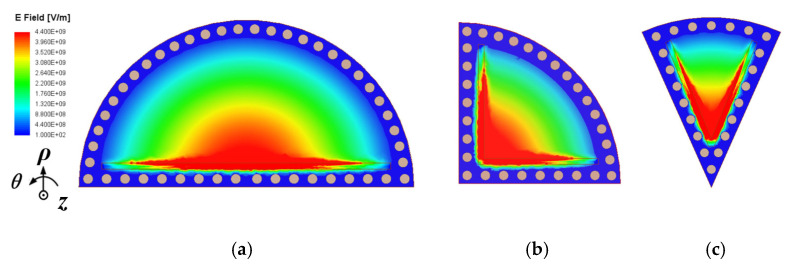
Configurations and electric-field distributions of the shielded SD-SMCSIW resonators: (**a**) SD-HMCSIW, (**b**) SD-QMCSIW, (**c**) SD-EMCSIW.

**Figure 4:**
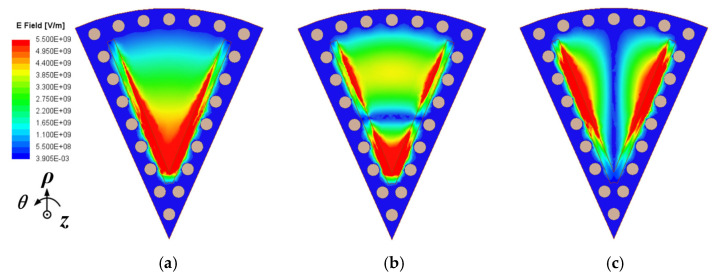
Electric-field distributions of the first three modes in SD-EMCSIW resonator: (**a**) TM_010_, (**b**) TM_020_, (**c**) TM_110_.

To mitigate the radiation loss caused by the traditional SIW open structure, a shielded substrate-integrated waveguide [25,30] configuration is considered, which utilizes a row of metallic via-holes with an open-edged slot. It functions as a magnetic wall to mimic the open edge found in conventional sub-mode CSIW resonant cavities. Thus, the configurations of the shielded SMCSIW (SD-SMCSIW) resonators are presented in Figure 3. This figure presents the SD-HMCSIW, SD-QMCSIW, and SD-EMCSIW resonators. The electric-field distributions are also demonstrated in Figure 1. It can be seen that the fundamental mode of the SD-SMCSIW is similar to the traditional SMCSIW. In this paper, the SD-EMCSIW cavity is selected as the basic unit resonator. The electric-field distributions of the first three modes in the SD-SMCSIW are plotted in Figure 4; it can be seen that the electric-field distributions of the SD-EMCSIW are similar to those of the unshielded sub-mode EMCSIW resonators. In addition, this series of resonators also exhibits similar properties. A SD-SMCSIW resonator utilizing a Taconic TLY-5 substrate with a relative permittivity (*ε_r_*) of 2.2, a thickness (*h*) of 0.508 mm, and a dissipation factor (tan *δ*) of 0.0009 can be examined by adjusting the angle *α* between 30 and 120 degrees. The eigenmode analysis method in HFSS can assist in analyzing the frequency relationships and changing trends of various resonance modes.

### 2.3. Analysis of Hybrid SD-EMCSIW and CSRR Resonator

**Figure 5:**
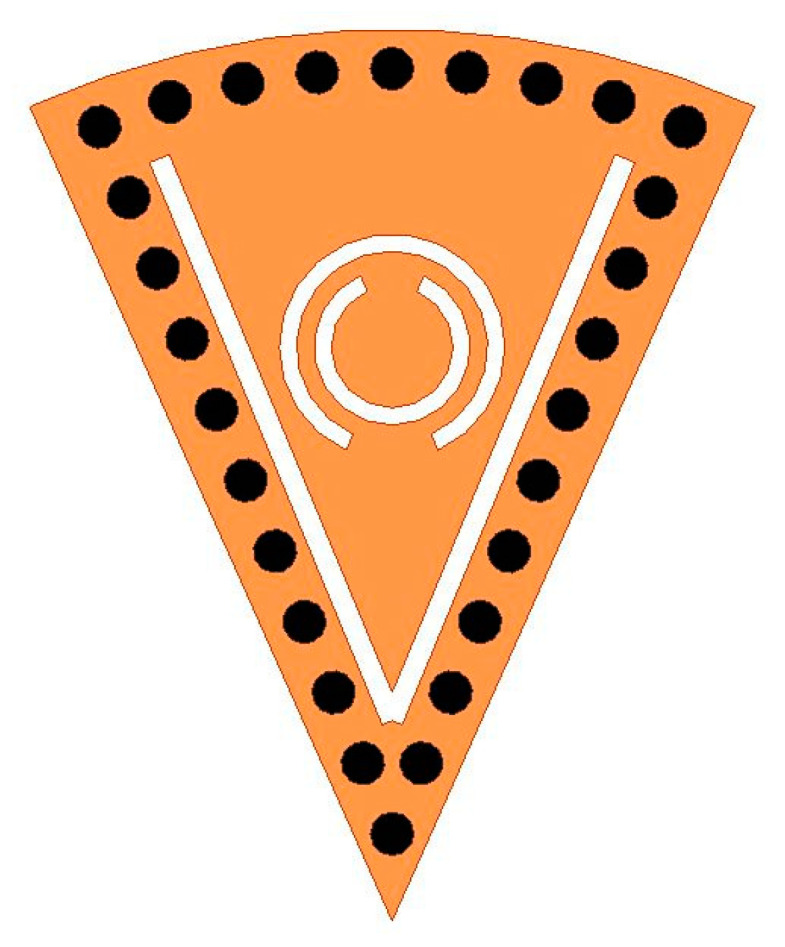
Configuration of the hybrid SD-EMCSIW and CSRR resonator.

To further minimize the dimension of the SD-EMCSIW resonator, CSRR resonators [31,32] are embedded into the SD-EMCSIW resonator to establish a new hybrid resonator. The structure of the hybrid SD-EMCSIW and CSRR resonator is presented in Figure 5. This resonator unit is selected as a potential choice for conducting the bandpass filter design. By utilizing the CSRR structure and integrating it into the SD-EMCSIW resonator, the resonance frequency of the fundamental mode shifts to the higher frequency band. In this instance, the occupied area of the hybrid SD-EMCSIW and CSRR resonator can be miniaturized. Meanwhile, the quality-factor of the hybrid shielded resonator can improve when utilizing the metallized via-holes to construct the shielding walls.

## 3. Design of the Proposed Hybrid Shielded Bandpass Filter

Based on the analysis of the various categories of the CSIW resonant cavities mentioned above, the hybrid SD-EMCSIW and CSRR resonator, with its merits of miniaturized physical size and high quality-factor, holds potential for designing passive components such as filters.

### 3.1. Design of SD-EMCSIW BPF with EM Coupling (Type I)

**Figure 6:**
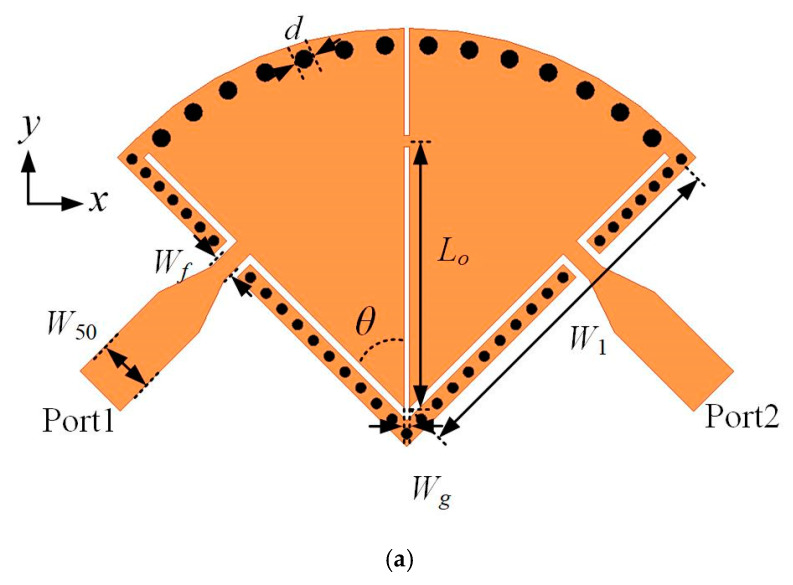

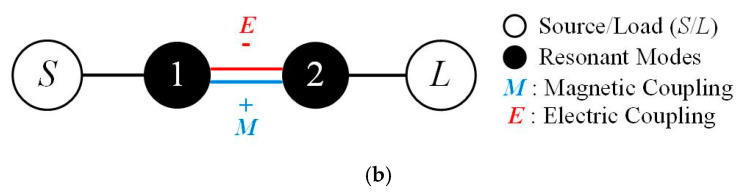
Configuration and coupling scheme of the second-order SD-EMCSIW BPF with EM coupling. (**a**) Structure of the second-order SD-EMCSIW BPF with mixed coupling. (**b**) Coupling scheme.

**Figure 7:**
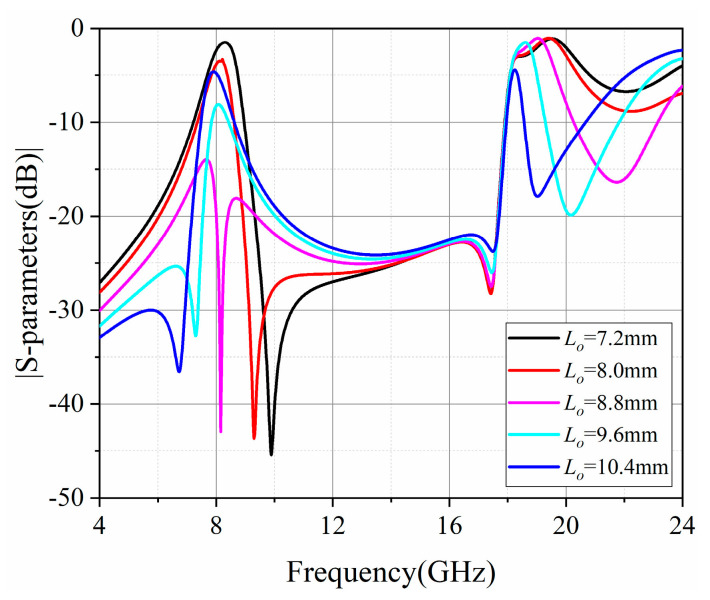
Simulated frequency responses of the second-order SD-EMCSIW BPF versus the location of offset microstrip line *L_o_*.

A second-order SD-EMCSIW bandpass filter (Type I) with EM coupling is depicted in Figure 6. It consists of two SD-EMCSIW resonators, a coupled microstrip line, and input/output feeding lines. The design parameters are listed as follows: *W*_50_ = 1.54 mm, *W*_1_ = 9.8 mm, *W_f_* = 0.5 mm, *W_g_* = 0.15 mm, *L_o_* = 7.2 mm, *θ* = 45°, and *d* = 0.5 mm. The coupling between two SD-EMCSIW resonators is electromagnetic (EM) coupling. The EM coupling can be controled by adjusting the connected microstrip line between two SD-EMCSIW resonators. The transmission responses of the second-order SD-EMCSIW BPF (Type I), with respect to the location of the connected microstrip line, are displayed in Figure 7. A transmission zero (TZ) can be generated near the operating passband. Furthermore, the transmission zero can be distributed by adjusting the microstrip stub to control the coupling strength of the filter. The position of the transmission zero can be derived using the following formulas [33,34]:

(4) $$f_{Z}=f_{0}\sqrt{\frac{M_{C}}{E_{C}}}$$

(5) $$k=\frac{M_{C}-E_{C}}{1-M_{C}E_{C}}$$

where *f_Z_* denotes the position of the transmission zero; *f*_0_ represents the center frequency; *k* demonstrates the coupling coefficients; and *M_c_* and *E_c_* characterize the magnetic coupling and electric coupling, respectively. As the position of the microstrip stub moves away from the center of the SD-EMCSIW resonant cavity, the transmission zero shifts from right side of the passband to the left side. Based on the formula mentioned above, when the mixed coupling is dominated by the magnetic coupling, the transmission zero will be distributed at the right side of the passband. While the mixed coupling is dominated by the electric coupling, the transmission zero will appear at the left side of the passband. In this instance, the transmission zero can be adjusted flexibly by tuning the position of the microstrip stub.

### 3.2. Design of Hybrid SD-EMCSIW and CSRR BPF with EM Coupling (Type II)

**Figure 8:**
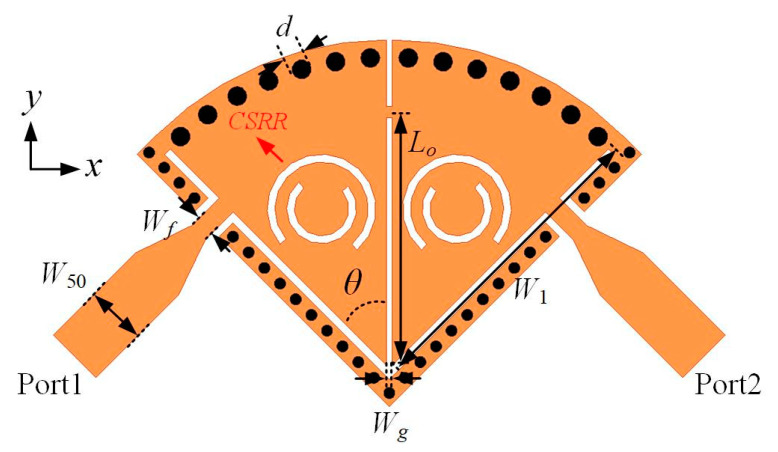
Configuration of the second-order hybrid SD-EMCSIW and CSRR BPF in Type II.

**Figure 9:**
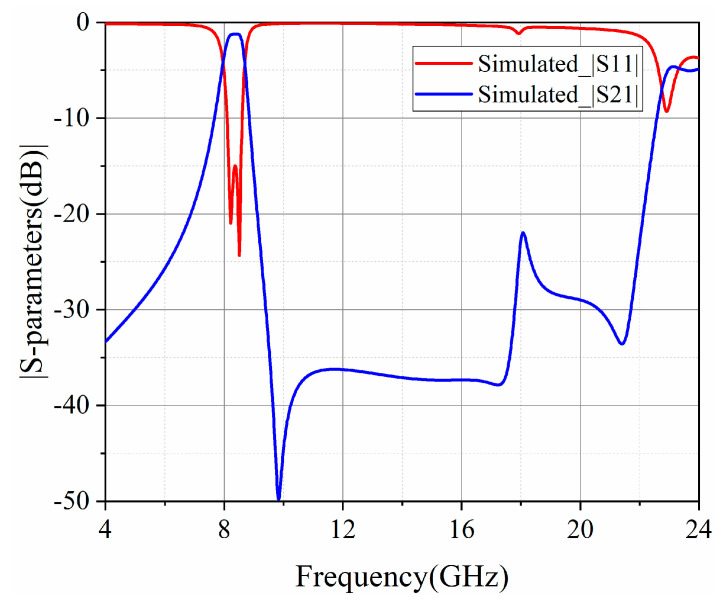
Frequency responses of the second-order hybrid SD-EMCSIW and CSRR BPF in Type II.

To miniaturize the physical dimension of the SD-EMCSIW BPF with EM coupling, the CSRR resonators are utilized in combination with the SD-EMCSIW resonant cavity. The configuration of the hybrid SD-EMCSIW and CSRR BPF with EM coupling (Type II) is demonstrated in Figure 8. The design parameters can be determined as follows: *W*_50_ = 1.54 mm, *W*_1_ = 7.9 mm, *W_f_* = 0.5 mm, *W_g_* = 0.15 mm, *L_o_* = 6.5 mm, *θ* = 45°, and *d* = 0.5 mm. The frequency responses of the second-order hybrid SD-EMCSIW and CSRR BPF (Type II) are exhibited in Figure 9. As shown, the center frequency of the second-order hybrid BPF is 8.4 GHz, with a 3 dB fractional-bandwidth (FBW) of 8.7%. The simulated insertion loss (IL) is 1.2 dB and the return loss (RL) is more than 15 dB over the working bandwidth. A transmission zero is generated at 9.84 GHz. Meanwhile, the spurious frequency at the upper stopband is generated at 2.66 *f*_0_, while the out-of-band rejection is more than 15 dB.

### 3.3. Improvement of Hybrid SD-EMCSIW and CSRR BPF with EM Coupling (Type III)

**Figure 10:**
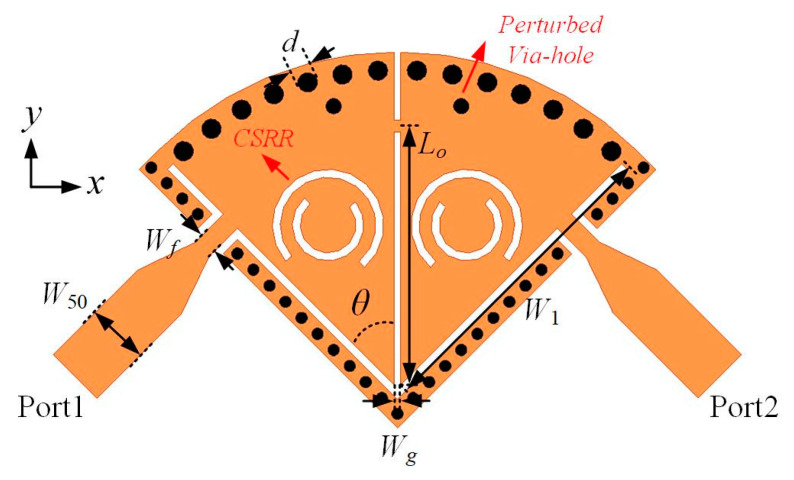
Configuration of the second-order hybrid SD-EMCSIW and CSRR BPF with extra perturbed via-holes in Type III.

**Figure 11:**
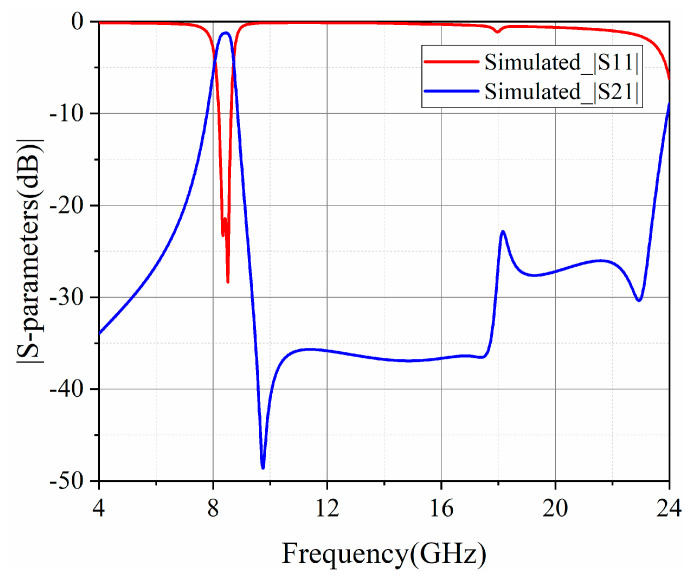
Frequency response of the second-order hybrid SD-EMCSIW and CSRR BPF with extra perturbed via-holes in Type III.

To improve the performance of the upper-stopband rejection further, a pair of metallized via-holes are introduced in the hybrid cavities to perturb the high-order modes. Thus, the spurious frequency at the upper stopband can be adjusted to the high frequency band. The setup of the hybrid SD-EMCSIW and CSRR BPF featuring EM coupling (Type III) is illustrated in Figure 10. The simulated frequency characteristics of the second-order hybrid SD-EMCSIW and CSRR BPF (Type III) are presented in Figure 11. As depicted, the central frequency of the second-order hybrid BPF is 8.4 GHz, with a 3 dB bandwidth of 650 MHz. The simulated IL features as 1.24 dB, and the RL exceeds 21.5 dB across the operational bandwidth. A transmission zero emerges at 9.76 GHz. Simultaneously, the spurious frequency in the upper stopband occurs at 2.82 *f_0_* as the spurious suppression is below 15 dB. By comparing Figure 9 and Figure 11, it can be observed that the suppression performance in Type III is better than Type II. Thus, it could be concluded that the extra metallized via-holes effectively improve the upper-stopband rejection.

## 4. Fabrication, Measurement, and Discussion

**Figure 12:**
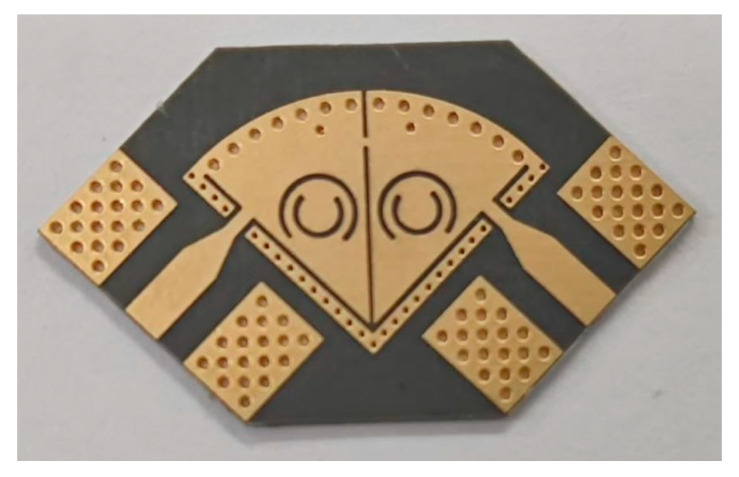
Photograph of the second-order hybrid SD-EMCSIW and CSRR BPF with mixed EM coupling scheme.

To verify the proposed design idea, a second-order hybrid SD-EMCSIW and CSRR bandpass filter with mixed electromagnetic coupling was fabricated by utilizing a single layer printed circuit board process. The Taconic TLY-5 substrate was adopted by using a thickness of 0.508 mm with a loss tangent of 0.0009. The photograph of the designed hybrid SD-EMCSIW and CSRR BPF is exhibited in Figure 12. The overall size of the proposed prototype is 13.5 mm × 13.0 mm (0.37λ_0_ × 0.36λ_0_).

**Figure 13:**
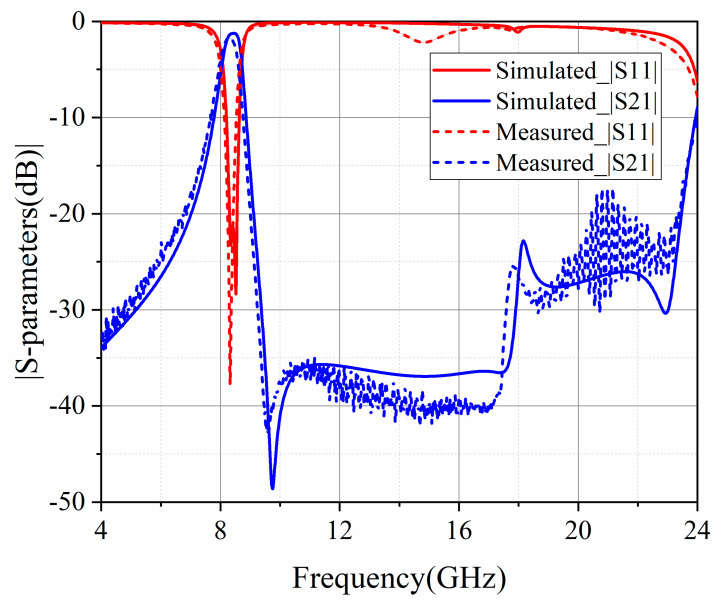
Simulated and measured frequency responses of the second-order hybrid SD-EMCSIW and CSRR BPF with mixed EM coupling scheme.

The measurement of the designed hybrid EMCSIW and CSRR BPF with a mixed electromagnetic coupling scheme was performed by vector network analyzer (VNA) Agilent Technologies N5244A (Santa Clara, CA, USA). The simulated and measured outcomes were provided in Figure 13. As observed, the center frequency of the fabricated second-order hybrid BPF is 8.3 GHz, with a 3 dB bandwidth of 8.1%. The simulated IL features as 1.73 dB, and the RL exceeds 10 dB across the operational bandwidth. It should be mentioned that the measured results contain the insertion loss of the testing connectors. The connectors soldered on the input and output ports are approximately 0.4 dB. In this situation, the real insertion loss is 1.33 dB after calibrating the testing connectors. A transmission zero emerges at 9.60 GHz. Simultaneously, the spurious frequency in the upper stopband occurs at 2.85 *f*_0_ as the suppression is below 15 dB. Moreover, the upper stopband can reach up to 2.53 *f*_0_ as the suppression is superior than 20 dB. From Figure 13, it can be concluded that the simulated and measured results reached a satisfactory agreement.

A comparison table between this work and other related publications in the literature is presented in Table 1. It can be observed that the proposed prototype utilizing the hybrid SD-EMCSIW and CSRR resonators exhibits the advantages of low insertion loss, excellent upper-stopband suppression, and miniaturized circuit size.

## 5. Conclusions

In this paper, a compact and wide-stopband bandpass filter, using hybrid SD-EMCSIW and CSRR resonators with a mixed electromagnetic coupling scheme, has been proposed and discussed. This hybrid BPF can generate a transmission zero near the passband to improve the frequency selectivity. Meanwhile, the transmission zero can be adjusted and positioned at the left or right side of the passband to satisfy the designed specification by controlling the strength of the electromagnetic coupling. The developed BPF exploits the superior features of low insertion loss, miniaturized physical dimensions, high shielding characteristics, and excellent upper-stopband rejection. Thus, the proposed hybrid SD-EMCSIW and CSRR BPF can be considered as a potential design choice for modern wireless communication and radar system applications.

## Figures and Tables

**Table 1:** Comparisons between this work and other published state-of-the-art research.

	Resonator Type	*f*_0_ (GHz)	IL (dB)	Number of TZs	Stopband Rejection	Circuit Size (*λ*_0_ × *λ*_0_)
[14]	QMSIW	4	1.02	1	>−20 dB up to 1.6 *f*_0_	0.79 × 0.54
[17]	QMSIW/EMSIW	8	0.9	1	>−23 dB up to 1.96 *f*_0_	0.71 × 0.44
[23]	Dual-Mode CSIW	8	2.15	2	Not Given	2.40 × 1.21
[24]	Perturbed CSIW	7.43	1.8	0	>−20 dB up to 1.48 *f*_0_	0.60 × 0.60
This work	Hybrid SD-EMCSIW/CSRR	8.3	1.33	1	>−20 dB up to 2.53 *f*_0_	0.37 × 0.36

## Data Availability

All data are included within the manuscript.
